# Bridging the Gap: The Role of Continuous Positive Airway Pressure Therapy in Diabetic Retinopathy Associated With Obstructive Sleep Apnea–A Perspective

**DOI:** 10.1002/hsr2.70502

**Published:** 2025-02-23

**Authors:** Sadia Ayoub, Hira Tanweer, Hafiza Aiman Javaid, Minahil Nasreen Khan, Shahzaib Ahmed, Mohammed Mahmmoud Fadelallah Eljack

**Affiliations:** ^1^ Jinnah Sindh Medical University Karachi Pakistan; ^2^ Isra University Hyderabad Pakistan; ^3^ Fazaia Ruth Pfau Medical College Karachi Pakistan; ^4^ Central Park Medical College Lahore Pakistan; ^5^ Department of Medicine Fatima Memorial Hospital College of Medicine and Dentistry Lahore Pakistan; ^6^ Community Department University of Bakht Alruda Ad Duwaym Sudan

**Keywords:** continuous positive airway pressure, CPAP, diabetes mellitus, diabetic retinopathy, obstructive sleep apnea

## Abstract

**Background:**

Obstructive sleep apnea (OSA) is a highly prevalent, yet under‐diagnosed sleep disorder and has a strong association with type 2 diabetes and diabetic retinopathy (DR). Vascular abnormalities, nocturnal glucose dysregulation, impaired blood flow, and hypoxia during OSA induce oxidative stress and promote the inflammatory pathways which increase the VEGF factor levels, leading to the progression of DR.

**Aims:**

To date, continuous positive airway pressure (CPAP) is the most effective, gold‐standard treatment for patients with moderate to severe OSA. However, the implications of CPAP for the treatment of DR due to OSA is still a topic of ongoing debate.

**Conclusion:**

Evidence suggests that the administration of CPAP therapy led to a reduction in retinal exudates and optical coherence tomography indices for retinal edema and also exhibited improvement in glycemic control, sleepiness, and overall health‐related quality of life. Nevertheless, there are limited studies present that have evaluated the impact of CPAP therapy on DR in patients with OSA and well‐designed studies are needed to confirm CPAP's therapeutic effect on DR despite these findings. Moreover, concerns regarding its long‐term safety, adherence challenges, and inconsistent study designs limit definitive conclusions about CPAP's efficacy in managing DR. This indicates the need for future studies to advocate for enhanced CPAP adherence strategies, refined diagnostic criteria for OSA, and large‐scale clinical trials to explore CPAP's therapeutic role in DR. Addressing these challenges could revolutionize clinical practices, optimize patient outcomes, and establish CPAP as a cornerstone in the integrated management of OSA and DR.

## 1 Introduction

Obstructive sleep apnea (OSA) is a chronic sleep disorder characterized by recurrent episodes of cessation in breathing (apnea) and overly shallow breathing (hyper apnea) during sleep due to mechanical resistance in the upper airway [1]. Approximately 936 million adults are known to suffer from moderate to severe OSA [2]. As reported by the American Academy of Sleep Medicine, OSA is a highly underdiagnosed and under‐reported sleep disorder as 80% of the OSA patients remain undiagnosed [2]. This condition is particularly prevalent among patients with type 2 diabetes mellitus [3], indicating an association between sleep disorders and metabolic disorders. Thus, patients experiencing insulin resistance or its complications are at increased risk for developing OSA. Diabetic retinopathy (DR) is the most common microvascular complication of diabetes, and it is also one of the leading causes of blindness in the diabetic population [4]. It is one of the most common retinal vascular diseases that affects every three in four patients experiencing diabetes for 15 years [5].

## 2 Association Between OSA and DR

Previous studies have reported a close association between OSA and DR [6, 7]. The sleep deprivation that occurs due to OSA affects different bodily functions including metabolic control. Studies show an association between poor sleep patterns and an increase in levels of HbA1c [8]. OSA is also associated with eye disorders like non‐arteritic anterior ischemic optic neuropathy (NAION), floppy eyelid syndrome (FES), keratoconus (KC), central serous retinopathy (CSR), and glaucoma. Therefore, ophthalmologists seeing patients with any of these conditions should consider screening and referring patients for assessment of possible OSA. In diabetic patients, abnormalities arise in microvasculature which causes stenosis of the vessels, eventually leading to ischemia. In diabetic patients, who often experience vascular abnormalities, nocturnal glucose dysregulation, impaired blood flow, and hypoxia, the intervals of systemic hypoxia during OSA will induce oxidative stress and promote the inflammatory pathways which will further increase the VEGF levels leading to the progression of DR [1]. Intermittent hypoxia triggers the release of pro‐inflammatory cytokines, including TNF‐α, IL‐8, and IL‐6 [9]. Intermittent hypoxia leads to have a stronger inflammatory effect, as repeated cycles of oxygen deprivation and reoxygenation contribute to the generation of reactive oxygen species and heightened oxidative stress [10]. Consequently, the hypoxic episodes associated with OSA may act as an additional driver of inflammation.

## 3 The Role of Continuous Positive Airway Pressure (CPAP) Therapy

To date, CPAP is the most effective, gold‐standard treatment for patients with moderate to severe OSA [11]. A CPAP machine is a device that delivers a continuous stream of pressurized air through a mask worn during sleep. This air stream at higher pressure prevents the airway from narrowing or collapsing while a person is asleep. This can reduce hypoxia‐related complications, which is beneficial for patients with DR. CPAP also helps in lowering blood pressure which plays a key role in managing OSA and DR. Although, CPAP is the preferred treatment for OSA, not all patients with OSA can tolerate CPAP. To address this issue, alternative modes of pressure delivery have been developed to enhance patient comfort and adherence. Auto‐adjustable positive airway pressure (APAP) devices can be used for the long‐term management of OSA and may also aid in the initial diagnosis and titration of conventional CPAP therapy [12].

Although, the main findings from Herth et al. indicated no significant effect of CPAP therapy on fasting blood glucose (FBG), HOMA‐IR, or fructosamine levels but revealed a statistically significant improvement in HbA1c and 24 h plasma glucose levels. Therefore, effectively treating OSA with CPAP could lead to a clinically meaningful reduction in HbA1c [13]. Nevertheless, the implications of CPAP for the treatment of DR due to OSA is still a topic of ongoing debate. In this article, we will try to establish an association between OSA and DR along with the effectiveness and safety of CPAP as a treatment for DR.

## 4 Current Evidence on the Efficacy of CPAP Therapy for DR in OSA Patients

A randomized control trial was conducted from 2016 to 2020, comprising a total of 83 patients that underwent CPAP with concomitant OSA and non‐proliferative DR. After a median follow‐up of 52 weeks, it was observed that administration of CPAP therapy led to a reduction in retinal exudates, optical coherence tomography indices for retinal edema and also exhibited improvement in glycemic control, sleepiness, and overall health‐related quality of life. Overall, it was observed that patients with OSA who undergo long‐term CPAP treatment have a slower progression of retinal disease. However, there was a higher incidence of retinal microhemorrhages reported with long‐term treatment with CPAP, indicating the need for future trials to validate the safety profile of CPAP for long‐term treatment [14].

Mason et al. reported that individuals with DR and co‐existing OSA who are compliant with CPAP therapy with ≥ 2.5 h per night for 6 consecutive months show improvement in visual acuity [15]. Furthermore, as reported in a retrospective study, high CPAP adherence decreases the progression to advanced DR and maculopathy [16].

However, findings from a meta‐analysis by Zhu et al. [17] suggested that OSA was significantly associated with an increased risk of DR. Sub‐group analysis on the type of diabetes mellitus was also performed, and it showed that OSA was linked to the risk of developing DR among both groups of patients with Type 1 and Type 2 diabetes mellitus. Furthermore, studies have reported that around 40%−80% of patients with type 2 diabetes mellitus have OSA. The rise in insulin resistance in patients with OSA increases the risk of ocular complications [18].

Nevertheless, there are limited studies present that have evaluated the impact of CPAP therapy on DR in patients with OSA and well‐designed studies are needed to confirm CPAP's therapeutic effect on DR despite these findings.

## 5 Current Limitations in Understanding CPAP's Role in DR

There is a need for better phenotyping of OSA patients. The current diagnostic criteria focus predominantly on anatomical factors, with minimal consideration for other pathophysiological traits, including arousal threshold, loop gain, and muscle tone. Additionally, the existing severity thresholds for OSA, more commonly known as the apnea‐hypopnea index (AHI), may not fully reflect the clinical impact of OSA. Therefore, revising severity thresholds can lead to more accurate assessment and management decisions. Moreover, patients affected with OSA may exhibit different symptoms and show varied treatment responses. Therefore, qualitative phenotyping incorporating anatomical and non‐anatomical traits is crucial to understanding distinct OSA subtypes and helps to optimize therapeutic strategies and enhance treatment outcomes [19].

Moreover, evidence regarding the importance of positive airway pressure therapy for OSA in improving multiple comorbidities is anecdotal. The long‐term impact of OSA on DR progression requires further investigation. Addressing this gap is essential for improving clinical management and patient outcomes. Moreover, there is a lack of robust clinical trials that have evaluated the efficacy of CPAP in DR. There is a general estimation of 4 h of CPAP usage per night but the evidence supporting this duration is limited [20]. Additionally, few studies reported adverse effects of CPAP such as increased IOP and superficial keratitis that still have not been thoroughly discussed.

Moreover, there was a lack of adherence to CPAP therapy in most of the studies [21]. The reasons for decreased adherence include lack of understanding of CPAP machine usage, claustrophobia, and CPAP mask fitting issues, hence the outcome of these studies could not reflect the actual benefits of CPAP in treating DR. Due to discomfort, pressure intolerance, and mask‐related issues, the number of hours a patient consistently uses CPAP varies [22]. Influence is implicated on adherence and overall therapeutic outcomes due to differences between nasal and full‐face masks [23]. Furthermore, the pressure settings required to maintain airway patency are not uniform across individuals, making standardization difficult [24].

Even though CPAP is effective in lowering AHI, improvement differs among patients, potentially influencing its downstream effects on metabolic and vascular parameters [25, 26]. Given this variability, future studies should stratify results based on CPAP adherence, pressure settings, and AHI reduction to better understand its role in DR.

## 6 Future Directions

There is limited evidence on the therapeutic potential of CPAP in preventing or delaying the progression of DR. Thus, it remains an area of interest and active research. Therefore, further clinical trials and studies should be conducted on a large sample size, with better compliance and frequent and long‐term follow‐ups to validate the efficacy of CPAP in patients with OSA and DR. Adherence with CPAP has been a consistent issue in almost all studies, without which, the results of the trials could not predict the actual effectiveness of CPAP. Therefore, future studies should ensure that participants enrolled in the trial receive adequate training on the usage of CPAP machines, mask fitting, and strategies to decrease discomfort associated with CPAP usage such as nasal congestion, aerophagia, and dry eyes. Furthermore, only those participants should be included in the intervention phase whose adherence and compliance were deemed optimal during the run in period, thereby yielding more accurate study outcomes.

A detailed study of the optimal hours of CPAP usage should also be conducted. In a recent review, Hanna and Kim explained 6 h of CPAP usage is optimal for better improvement opposing the previous 4 h criteria for compliance and they also suggested the percentage of total sleep/individualized optimal hours is the best predictor of compliance for an individual. However, these findings need to be thoroughly followed.

OSA is an under‐diagnosed condition due to patients ignoring symptoms until they become severe, thereby decreasing the reporting to the physician and decreasing screening since not all patients exhibit similar symptoms, and the severity of the symptoms also varies among patients. Similarly, DR is a silent disease until it progresses to blindness. For better treatment outcomes and prevention of OSA‐related complications, it is crucial to screen all diabetic patients visiting the clinics for any of the symptoms related to OSA and refer them for DR evaluation for early diagnosis and initiation of treatment before the complications begin.

## 7 Conclusion

In conclusion, the intricate relationship between OSA and DR highlights a pressing need for further exploration of the therapeutic potential of CPAP for the management of DR. However, the current evidence reveals significant gaps in understanding, and definitive conclusions remain elusive. This calls for the need for comprehensive, well‐designed clinical trials with larger sample sizes, improved adherence of the participants, and longer and more structured follow‐ups to validate the effectiveness of CPAP therapy for OSA‐linked DR. Addressing these gaps could transform the clinical practice and pave the way for more effective treatment strategies, thereby enhancing the patient outcomes.

## Author Contributions

**Sadia Ayoub:** conceptualization, writing–original draft, writing–review and editing. **Hira Tanweer:** conceptualization, writing–original draft. **Hafiza Aiman Javaid:** writing–original draft. **Minahil Nasreen Khan:** writing–original draft. **Shahzaib Ahmed:** writing–review and editing. **Mohammed Mahmmoud Fadelallah Eljack:** supervision, writing–review and editing. All authors have read and approved the final version of the manuscript.

## Ethics Statement

The authors have nothing to report.

## Conflicts of Interest

The authors declare no conflicts of interest.

## Transparency Statement

The lead author, Mohammed Mahmmoud Fadelallah Eljack, affirms that this manuscript is an honest, accurate, and transparent account of the study being reported, that no important aspects of the study have been omitted, and that any discrepancies from the study as planned (and if relevant, registered) have been explained.

## Data Availability

Data sharing is not applicable to this article as no data sets were generated or analyzed during the current study.
